# Prevalence of hepatitis C virus and human immunodeficiency virus in a group of patients newly diagnosed with active tuberculosis in Porto Alegre, Southern Brazil

**DOI:** 10.1590/0074-02760160352

**Published:** 2017-04

**Authors:** Cintia Costi, Tarciana Grandi, Maria Laura Halon, Márcia Susana Nunes Silva, Cláudia Maria Dornelles da Silva, Tatiana Schäffer Gregianini, Lia Gonçalves Possuelo, Carla Adriane Jarczewski, Christian Niel, Maria Lucia Rosa Rossetti

**Affiliations:** 1 Secretaria Estadual da Saúde do Rio Grande do Sul, Fundação Estadual de Produção e Pesquisa em Saúde, Centro de Desenvolvimento Científico e Tecnológico, Porto Alegre, RS, Brasil; 2 Universidade Luterana do Brasil, Canoas, RS, Brasil; 3 Secretaria Estadual da Saúde do Rio Grande do Sul, Fundação Estadual de Produção e Pesquisa em Saúde, Instituto de Pesquisas Biológicas, Laboratório Central do Estado, Porto Alegre, RS, Brasil; 4 Universidade de Santa Cruz do Sul, Programa de Pós-Graduação em Promoção da Saúde, Santa Cruz do Sul, RS, Brasil; 5 Secretaria Estadual da Saúde do Rio Grande do Sul, Hospital Sanatório Partenon, Porto Alegre, RS, Brasil; 6 Fundação Oswaldo Cruz-Fiocruz, Instituto Oswaldo Cruz, Laboratório de Virologia Molecular, Rio de Janeiro, RJ, Brasil

**Keywords:** HCV, tuberculosis, coinfection, genotypes, HIV, Southern Brazil

## Abstract

**BACKGROUND:**

Porto Alegre is the Brazilian state capital with second highest incidence of tuberculosis (TB) and the highest proportion of people infected with human immunodeficiency virus (HIV) among patients with TB. Hepatitis C virus (HCV) infection increases the risk of anti-TB drug-induced hepatotoxicity, which may result in discontinuation of the therapy.

**OBJECTIVES:**

The aim of this study was (i) to estimate prevalence of HCV and HIV in a group of patients newly diagnosed with active TB in a public reference hospital in Porto Alegre and (ii) to compare demographic, behavioural, and clinical characteristics of patients in relation to their HCV infection status.

**METHODS:**

One hundred and thirty-eight patients with TB were tested for anti-HCV antibody, HCV RNA, and anti-HIV1/2 antibody markers. HCV RNA from real-time polymerase chain reaction (PCR)-positive samples was submitted to reverse transcription and PCR amplification. The 5′ non-coding region of the HCV genome was sequenced, and genotypes of HCV isolates were determined.

**FINDINGS:**

Anti-HCV antibody, HCV RNA, and anti-HIV antibodies were detected in 27 [20%; 95% confidence interval (CI), 13-26%], 17 (12%; 95% CI, 7-18%), and 34 (25%; 95% CI, 17-32%) patients, respectively. HCV isolates belonged to genotypes 1 (n = 12) and 3 (n = 4). Some characteristics were significantly more frequent in patients infected with HCV. Among them, non-white individuals, alcoholics, users of illicit drugs, imprisoned individuals, and those with history of previous TB episode were more commonly infected with HCV (p < 0.05).

**MAIN CONCLUSIONS:**

HCV screening, including detection of anti-HCV antibody and HCV RNA, will be important to improving the management of co-infected patients, given their increased risk of developing TB treatment-related hepatotoxicity.

Tuberculosis (TB), an infectious disease caused by *Mycobacterium tuberculosis,* remains a major public health problem in many countries. In Brazil, the incidence of TB was estimated at 42-46 cases per 100,000 people/year (WHO 2015a). Porto Alegre, a city located in Southern Brazil, is the state capital with the second highest (89/100,000) TB incidence in the country (MS 2016). The ongoing HIV/AIDS pandemic is one of the greatest challenges facing TB control, because HIV-induced immunosuppression increases the risk of latent TB activation considerably (Braun et al. 1993). In Brazil, about 10% of patients with TB are coinfected with HIV. In Porto Alegre, however, that proportion reaches 25% (MS 2016). Simultaneous administration of multiple antimicrobial agents has proved to be highly effective in treating TB. In Brazil, the anti-TB regimen is composed of rifampicin, isoniazid, and pyrazinamide (RHZ). In 2010, the Ministry of Health added ethambutol to the therapeutic guidelines. However, all anti-TB regimens are known to cause hepatotoxicity (Ramappa & Aithal 2013).

Similar to TB, hepatitis C virus (HCV) infection is an important global health issue, with 130-150 million people infected worldwide. Approximately, 15-45% of infected persons spontaneously clear the virus within six months of infection. The remaining 55-85% of infected persons develops chronic HCV infection, which, in some cases, leads to liver cirrhosis within 20 years (WHO 2015b). Detection of HCV RNA in the sera of anti-HCV-positive people by reverse transcription-polymerase chain reaction (RT-PCR) allows virus carriers (HCV RNA-positive people) to be distinguished from individuals who were infected but have cleared the virus (HCV RNA-negative people). HCV shows high genetic variability, and seven main HCV genotypes (1-7) have been identiﬁed. Genotypes 1 and 3, in this order, are the most prevalent in Brazil (Campiotto et al. 2005, da Silva et al. 2007, Lampe et al. 2013).

Hepatotoxicity is a major complication of anti-TB treatment that may result in therapy discontinuation. For this reason, liver function is tested before treatment. Although HCV coinfection has been shown to increase the risk of anti-TB drug-induced hepatotoxicity (Lomtadze et al. 2013, Kim et al. 2016) and death (Bushnell et al. 2015), patients without symptoms of liver disease are not systematically tested for anti-HCV antibody and HCV RNA before beginning anti-TB therapy.

Considering that most chronic HCV carriers and many HIV-infected people are unaware of their condition, the aims of this study were (i) to evaluate the prevalence of anti-HCV antibody, HCV RNA, and anti-HIV markers in a group of patients newly diagnosed with active TB in a public hospital of Porto Alegre, and (ii) to compare demographic, behavioural, and clinical characteristics of the patients in relation to their HCV infection status.

## MATERIALS AND METHODS

*Samples* - Plasma samples were collected in a previous study (Possuelo et al. 2008) from patients attending the outpatient section of Sanatório Partenon Hospital, a public TB reference hospital located in Porto Alegre, Rio Grande do Sul, Brazil. At the time of blood collection (August 2005-June 2007), patients provided demographic, clinical, and epidemiological data in response to a questionnaire, as well as written informed consent for use of their samples in more than one study. In 2013, the Research Ethics Committee of Fundação Estadual de Produção e Pesquisa em Saúde approved the use of these samples for the present study (registration number CAAE 13878213.0.0000.5320).

All patients were adults (> 18 years), newly diagnosed with active TB, and ready to start daily treatment with RHZ, as recommended by the Brazilian National Tuberculosis Program at the time of sample collection. Active TB was diagnosed after a complete medical evaluation that included clinical examination, chest radiograph, sputum smear microscopy, and mycobacterial culture. Out of a total of 669 patients examined, the 138 patients included in this study were those who met all three requirements: (i) individuals whose hepatitis B surface antigen (HBsAg), anti-HCV antibody, and anti-HIV1/2 antibody test results were available, (ii) individuals who did not show clinical signs of liver disease, and (iii) individuals who did not show elevated levels of transaminases or bilirubin (more than twice the upper limit), as measured by standard laboratory tests.

*Viral RNA extraction, reverse transcription, and cDNA amplification* - All 138 plasma samples (aliquots of 150 µL stored at -70ºC) were submitted to RNA extraction using a Nucleospin RNA Virus kit (Macherey-Nagel, Düren, Germany) according to the manufacturer’s instructions. After extraction, RNA was resuspended in 30 µL of water, and 40 units of RNaseOUT Ribonuclease Inhibitor (Invitrogen, Carlsbad, CA) was added. RNA was stored at -70ºC until use.

HCV RNA (13 µL) was submitted to reverse transcription, and the 3′-X-tail element of the viral genome was amplified by real-time PCR performed on a final volume of 30 µL using a Superscript III Platinum One Step qRT-PCR Kit (Invitrogen) with previously described (Drexler et al. 2009) oligonucleotide primers XTF5 and HCMgR2 (670 nM each) and HCV-specific probe HCVMGB2 (170 nM). RT-PCR was performed using a 7500 Real-Time PCR System (Applied Biosystems, Foster City, CA) at 50ºC for 30 min and 95ºC for 2 min, followed by 55 cycles at 95ºC for 20 s and 55ºC for 45 s. The limit of detection was 50 IU/mL. All assays were performed in duplicate.

*HCV genotyping* - HCV RNAs extracted from samples that were positive by real-time PCR were amplified by conventional reverse transcription-PCR using a Superscript One-Step RT-PCR System with Platinum *Taq* DNA polymerase (Invitrogen). This reaction was performed with 13 µL of RNA in a final volume of 50 µL. The oligonucleotide primers HCV1 and HCV2 used in the reaction (600 nM each) were described previously (Krug et al. 1996). After RT at 45ºC for 30 min, PCR amplification was performed with 55 cycles at 94ºC for 30 s, 55ºC for 30 s, and 72ºC for 30 s, followed by a final elongation for 5 min at 72ºC.

Amplicons (260-bp fragments of the conserved 5′ non-coding region of the HCV genome) were purified with a PureLink PCR Purification Kit (Life Technologies, Carlsbad, CA). Nucleotide sequencing was carried out in both directions using a BigDye Terminator v3.1 Cycle Sequencing Kit (Applied Biosystems), and sequencing reactions were performed in a 3130xl Genetic Analyzer (Applied Biosystems).

HCV genotypes were determined using the Basic Local Alignment Search Tool (BLAST) algorithm (http://blast.ncbi.nlm.nih.gov/Blast.cgi), which calculates the percent homology between a given nucleic acid query sequence and sequences of HCV isolates of known genotypes in the database.

*Statistical analysis* - Qualitative variables were compared using a χ^2^ test or Fisher’s exact test as appropriate. Quantitative variables were analysed using a Student’s *t*-test or Mann-Whitney *U* test (two groups) for normally and non-normally distributed data, respectively.

All analyses were performed using SPSS version 12.0 software for Windows (SPSS Inc., Chicago, IL). All statistical tests were evaluated at a 0.05 significance level.

## RESULTS

Of 138 serum samples, 27 [20%; 95% confidence interval (CI), 14-27%] were anti-HCV positive. HCV RNA was detected in 14 of these samples, as well as in three of the 111 anti-HCV negative samples (Figure). Thus, a total of 17 (12%, 95% CI, 8-19%) patients were HCV RNA positive. HCV genotypes of 16/17 (94%) RNA samples were determined. Twelve belonged to genotype 1, and four belonged to genotype 3. In addition, 34/138 (25%) patients were tested positive for anti-HIV.

**Figure:**
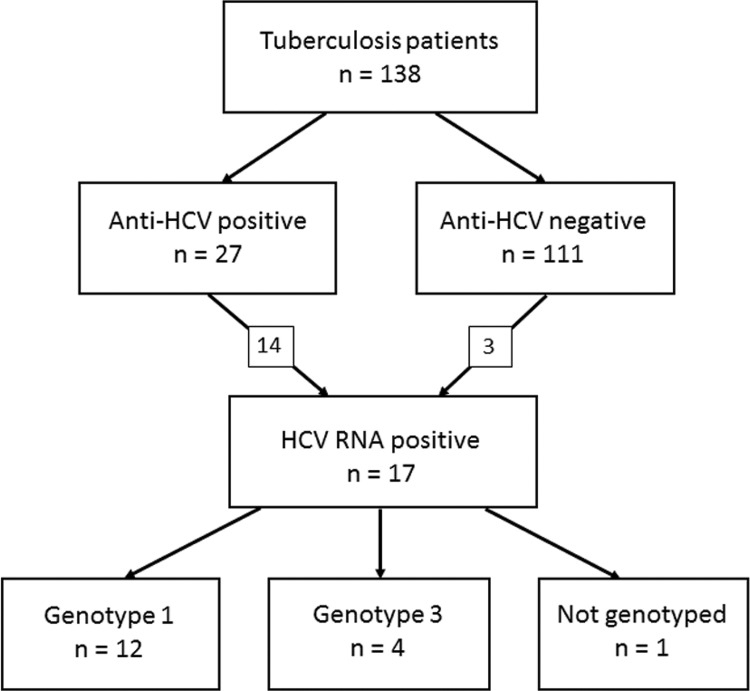
Detection of anti-hepatitis C virus (HCV) antibodies and HCV RNA in serum samples of Brazilian patients with tuberculosis and HCV genotypes.

Table shows the demographic, behavioural, and clinical characteristics of the patients with TB under study. Patients were 18-75 years old (mean ± SD, 38.0 ± 12.9 years). Two-thirds (67%; 95% CI, 59-75%) were males, 57% (95% CI, 49-65%) were whites, and 25% (95% CI, 18-32%) were coinfected with HIV. In a comparison of patients positive for anti-HCV antibodies and/or HCV RNA (n = 30) and anti-HCV antibody- and HCV RNA-negative subjects (n = 108), a significantly higher proportion (60% vs. 38%) of non-white people was observed in the first group. Illicit drug use, alcohol abuse, history of incarceration, current pulmonary TB, and a previous TB episode were characteristics that were significantly more frequent in HCV-positive patients than in those that were HCV negative. Furthermore, the proportion of HIV-infected patients was two-fold higher among HCV-positive patients (40%; 95% CI, 25-58%) than among HCV-negative patients (20%; 95% CI, 14-29%) (p < 0.05). As expected, mean alanine aminotransferase (ALT) and aspartate aminotransferase (AST) levels were only slightly higher in the HCV-positive patients (Table), because patients with evidence of chronic liver disease were excluded from the study.

**TABLE:** Demographic, behavioural, and clinical characteristics of patients with tuberculosis and a comparison of characteristics between patients positive for hepatitis C virus (HCV) by one infection marker or negative by both

Feature	Total (n = 138)	HCV infection markers (anti-HCV and HCV RNA)		

		At least one (n = 30)	Both negative (n = 108)	p value
Demographic data				
Age, M ± SD (years)	38.0 ± 12.9	41.1 ± 10.1	37.2 ± 13.5	NS
Sex (male)	93 (67%)	24 (80%)	69 (64%)	NS
Skin colour				
White	79 (57%)	12 (40%)	67 (62%)	< 0.05
Non white	59 (43%)	18 (60%)	41 (38%)	
Behaviour				
Cohabiting with partner	74 (54%)	18 (64%)	55 (51%)	NS
Illicit drug use	29 (21%)	12 (40%)	17 (16%)	< 0.05
Alcohol abuse	18 (13%)	11 (37%)	7 (7%)	< 0.001
Tobacco use	64 (47%)	18 (56%)	46 (43%)	NS
History of incarceration	10 (7%)	5 (17%)	5 (5%)	< 0.05
Tuberculosis				
Pulmonary tuberculosis	117 (85%)	30 (100%)	87 (81%)	< 0.05
Extrapulmonary tuberculosis	21 (15%)	0 (0%)	21 (20%)	
History of previous tuberculosis	26 (19%)	10 (33%)	16 (15%)	< 0.05
Laboratory data				
Anti-HIV1/2 positive	34 (25%)	12 (40%)	22 (20%)	< 0.05
HBsAg positive	1 (1%)	1 (4%)	0 (0%)	NS
ALT, M ± SD (IU/L)	28.7 ± 18.9	30.7 ± 19.1	28.1 ± 18.9	NS
AST, M ± SD (IU/L)	28.6 ± 18.2	35.0 ± 20.4	26.8 ± 17.2	< 0.05
Total bilirubin, M ± SD (mg/dL)	0.43 ± 0.33	0.50 ± 0.54	0.42 ± 0.24	NS

NS: not significant.

## DISCUSSION

An anti-HCV prevalence of 1.1-1.6% has been estimated in the urban Brazilian population (Pereira et al. 2013). In 2010, Porto Alegre reported the second highest hepatitis C rate (40.4 cases/100,000 inhabitants) among the Brazilian state capitals (MS 2012). Anti-HCV prevalence rates are generally higher among patients with TB than in the general population. In this study, 27/138 (20%) adult patients newly diagnosed with TB tested anti-HCV positive. This proportion was comparable to rates (12-22%) previously reported in Argentina (Pando et al. 2008) and Georgia (Richards et al. 2006, Lomtadze et al. 2013) and significantly higher than that (7%) reported in Taiwan (Wang et al. 2011).

Most studies on TB-HCV coinfection have used anti-HCV antibody as the sole marker of infection, without testing for HCV RNA. However, testing for both markers, as in this study, is more appropriate, because the anti-HCV positive/HCV RNA-negative pattern is common, due to high (15-45%) rates of spontaneous HCV clearance (WHO 2015b). This pattern is expected to increase in the near future because of the advent of new HCV therapies that completely eliminate the virus (Pawlotsky 2014). Although less common, anti-HCV-negative/HCV RNA positive results can be observed either in very recent infections (immunological window period) or with immunodeficiency. For example, in this study, one of the three anti-HCV negative/HCV RNA-positive patients was infected with HIV. When anti-HCV antibodies are not detectable, the use of molecular techniques to recognise HCV RNA are required (Kamili et al. 2012, Firdaus et al. 2015).

The regimen currently used to treat patients with TB has hepatotoxic effects, which can be compounded with HCV coinfection. Because most chronic HCV carriers and many HIV-infected people are unaware of their condition, the aims of this study were (i) to evaluate the prevalences of HCV and HIV infection in a group of patients newly diagnosed with active TB in a public reference hospital in Porto Alegre, Brazil, and (ii) to compare demographic, behavioural, and clinical characteristics of patients in relation to the HCV status. The present study differed from a previous one performed in the same hospital a few years earlier (Nader et al. 2010). In this study, we tested for viral RNA, which allowed us to distinguish ‘true’ HCV carriers from people who had been infected but cleared the virus. HCV RNA was thus detected in 14 of 27 anti-HCV positive patients, as well as in three of 111 anti-HCV-negative ones. The proportion of patients with TB that were HCV carriers (17/138, 12%) was about eight times higher than that observed in the urban Brazilian population, despite the fact that patients with elevated serum transaminase levels were excluded from this study. Such high rates of HCV may be clinically significant, considering that the incidence of abnormal liver function tests (and even mortality) with anti-TB treatment is higher in HCV-infected individuals than in the non-infected (Nader et al. 2010, Lomtadze et al. 2013, Mo et al. 2014).

HCV coinfection was associated with skin colour, illicit drug use, alcohol abuse, history of incarceration, previous TB, and anti-HIV1/2 positivity (Table). Some of these associations, such as the use of illicit drugs (Sirinak et al. 2008), history of incarceration (Richards et al. 2006), and HIV infection (Reis et al. 2011), have been observed previously in Asia and the Americas.

In Buenos Aires, Argentina, anti-HCV and anti-HIV prevalences of 12% and 17%, respectively, were determined in a group of 205 patients with TB (Pando et al. 2008). In another study from Central Brazil, the corresponding proportions were 7.5% and 28%, respectively (Reis et al. 2011). Although the proportion of anti-HIV-positive patients (25%) was similar to that in the studies cited above, the rate of anti-HCV-positive individuals (20%) was higher in this study. This may be explained by regional variations in HCV prevalence. Indeed, as previously mentioned, Porto Alegre presents one of the highest rates of hepatitis C among the Brazilian state capitals (MS 2012).

The prevalence of HIV infection was twice as high in TB/HCV-coinfected patients (40%) as in patients infected with TB only (20%) (Table). This result was in agreement with those of recent studies performed in Brazil (Reis et al. 2011) and China (Mo et al. 2014). In Thailand, a study revealed a very high (31%) anti-HCV prevalence among HIV/TB-coinfected patients (Sirinak et al. 2008). In all cases, high rates of TB, HCV, and HIV coinfection are likely a result of the high proportion (21% in this study) of injecting drug users in the populations under study (Friedland 2010).

According to the World Health Organization (WHO 2016), in 2014, at least one-third of people living with HIV worldwide were infected with TB bacteria. At the same time, the number of cases of hepatitis C infection and related diseases increased over the last two decades (Lozano et al. 2012). However, the effect of chronic hepatitis C on patients with TB or coinfected with TB and HIV has not been evaluated well. HCV infection is not a contraindication for anti-TB treatment, which is administered as a standard regimen. However, the monitoring schedule may change, since such patients may require closer laboratory monitoring than those who are uninfected. HCV screening, including anti-HCV antibody and HCV RNA detection, will be important to improving the management of coinfected patients, given their increased risk of developing hepatotoxicity as a consequence of anti-TB treatment. We therefore suggest routine HCV screening, as commonly performed for HIV, in places with known high HCV prevalences.

The fact that all of the patients in this study were from the same hospital was a limitation of the study. However, the sample was representative of the larger population because the hospital is a reference centre for TB and drug-resistant TB cases, and it is located in a region that serves a population of 180,000, with pockets poverty and indigence among them. Another limitation is the small number of patients included in the study. Despite these limitations, data presented here can contribute to addressing the TB-HCV coinfection problem in Brazil and may help public health authorities to make decisions related to screening of patients and selection of treatment protocols.
